# Efficient SNN multi-cores MAC array acceleration on SpiNNaker 2

**DOI:** 10.3389/fnins.2023.1223262

**Published:** 2023-08-07

**Authors:** Jiaxin Huang, Florian Kelber, Bernhard Vogginger, Chen Liu, Felix Kreutz, Pascal Gerhards, Daniel Scholz, Klaus Knobloch, Christian G. Mayr

**Affiliations:** ^1^Infineon Technologies Dresden, Dresden, Germany; ^2^Highly-Parallel VLSI-Systems and Neuro-Microelectronics, Faculty of Electrical and Computer Engineering, Institute of Principles of Electrical and Electronic Engineering, Technische Universität Dresden, Dresden, Germany; ^3^Centre for Tactile Internet with Human-in-the-Loop (CeTI), Cluster of Excellence, Technische Universität Dresden, Dresden, Germany

**Keywords:** SpiNNaker 2, SNN, MAC array, SpGEMM, multi-core load balancing deployment

## Abstract

The potential low-energy feature of the spiking neural network (SNN) engages the attention of the AI community. Only CPU-involved SNN processing inevitably results in an inherently long temporal span in the cases of large models and massive datasets. This study introduces the MAC array, a parallel architecture on each processing element (PE) of SpiNNaker 2, into the computational process of SNN inference. Based on the work of single-core optimization algorithms, we investigate the parallel acceleration algorithms for collaborating with multi-core MAC arrays. The proposed Echelon Reorder model information densification algorithm, along with the adapted multi-core two-stage splitting and authorization deployment strategies, achieves efficient spatio-temporal load balancing and optimization performance. We evaluate the performance by benchmarking a wide range of constructed SNN models to research on the influence degree of different factors. We also benchmark with two actual SNN models (the gesture recognition model of the real-world application and balanced random cortex-like network from neuroscience) on the neuromorphic multi-core hardware SpiNNaker 2. The echelon optimization algorithm with mixed processors realizes 74.28% and 85.78% memory footprint of the original MAC calculation on these two models, respectively. The execution time of echelon algorithms using only MAC or mixed processors accounts for ≤ 24.56% of the serial ARM baseline. Accelerating SNN inference with algorithms in this study is essentially the general sparse matrix-matrix multiplication (SpGEMM) problem. This article explicitly expands the application field of the SpGEMM issue to SNN, developing novel SpGEMM optimization algorithms fitting the SNN feature and MAC array.

## 1. Introduction

Coupling spatial and temporal information, the SNN shows promise in simulating biologically related models more comprehensively and efficiently. The CPU-based system is widely used for simulating these brain-inspired neural networks by taking advantage of flexibility. However, the efficient input spike encoding way is still in the exploration stage, and a gap still exists between the current encoding efficiency and that of the human brain, which reduces the expected sparsity of the input signal and extends the CPU running time. Moreover, to accommodate the serial operation mechanism, the model needs to introduce additional information when deployed to the hardware, such as the storage address of neurons, extra memory occupation owing to non-equivalent connections, and intermediate state storage buffers. This not only burdens the memory space but also inevitably requires more time to execute the corresponding pre- and post-neuron matching algorithm for information transfer and neural update, which is detrimental to the operation of real-time SNN inference.

To address these issues caused by pure CPU systems, we introduced the parallel computing concept into SNN inference. The feasibility of parallel architecture processing SNN lies in the neurons of SNN being typically governed by the same type of equations (Yavuz et al., 2016). As a result, the single-instruction-multiple-data (SIMD) architecture of MAC fits SNN calculation. This study targets a wholly parallel calculation based on the more efficient matrix parallelism. We emulate the SNN inference on SpiNNaker 2 (Mayr et al., 2019), which integrates the MAC array in each processing element (PE). The parallelism of this integrated hardware component has the potential of speeding up SNN inference in a sufficiently parallel manner. Nevertheless, there are two challenges to tackle:

- Memory alignment: The memory alignment for catering to the MAC array architecture triggers an issue of data volume surges, blocking the possibility of deploying more neurons and synapses on limited hardware resources.
- Multi-core distribution: The unconsidered multi-core distribution of the large-scale model can differentiate the spatial-temporal overhead among activated PEs, wasting the resources in space and time and affecting the performance of applications with strict requirements.

This study addresses these two challenges by lossless densifying the memory-aligned model information and splitting matrix multiplication operands into multiple PEs in a spatial-temporal load-balancing way.

Essentially, accelerating SNN inference with our algorithms is the SpGEMM problem, as explained in Section 2.2. SpGEMM is very popular in high-performance computing, mainly used in algebra and graph analysis (Gao et al., 2020). The vast majority of the relevant studies, such as Davis (2018), Zhang et al. (2020), and An and Çatalyürek (2021), are based on the “row-wise” algorithm proposed by Gustavson (1978), also known as compressed sparse row format (CSR) or Yale sparse matrix format. This traditional algorithm is unsuitable for using MAC array accelerating SNN, so we propose a brand-new optimization algorithm set, which can accelerate the SNN processing when alleviating the ineffective memory footprint. This algorithm set, consisting of four algorithms up to now, provides an alternative to the traditional method for solving the SpGEMM problem. To the best of our best knowledge, our work is the first to build a bridge between the concept of SpGEMM and SNN, expand the application field of SpGEMM to SNN, and tackle the SNN inference using the MAC array with new SpGEMM algorithms.

As a follow-up to our previous study that states three algorithms of information densification (Huang et al., 2023), this study proposes Echelon Reorder, filling in the unoptimized aspects of that work, completing the optimization algorithm set to fully resist the data sparsity caused by the SNN characteristics and fixed MAC array hardware structure. The corresponding splitting and deployment strategies proposed in this study extend the application range of the whole optimization algorithm set from single PE to multi-core, enabling accelerating the larger model on SpiNNaker 2 effectively. Furthermore, the compact splitting strategy fully uses each PE's memory resource, paving the way for the subsequent high-performance multiple tasks deployment on this multi-core neuromorphic platform.

This study briefly introduces the hardware and software cornerstones in Section 2. Then, based on them, we elaborate on the Echelon Reorder algorithm for weight and input pure and mixture processor splitting strategies and also multi-core role-based SNN model deployment in Section 3. Next, Section 4 evaluates the performance of this proposed processing chain. Finally, we conclude this article in Section 5.

## 2. Prerequisite

This section provides the hardware and software foundations for the next section concerning the MAC array architecture of SpiNNaker 2 and the stacked matrix-multiplication operands essential for accelerating SNN inference.

### 2.1. MAC array

**Figure 1:**
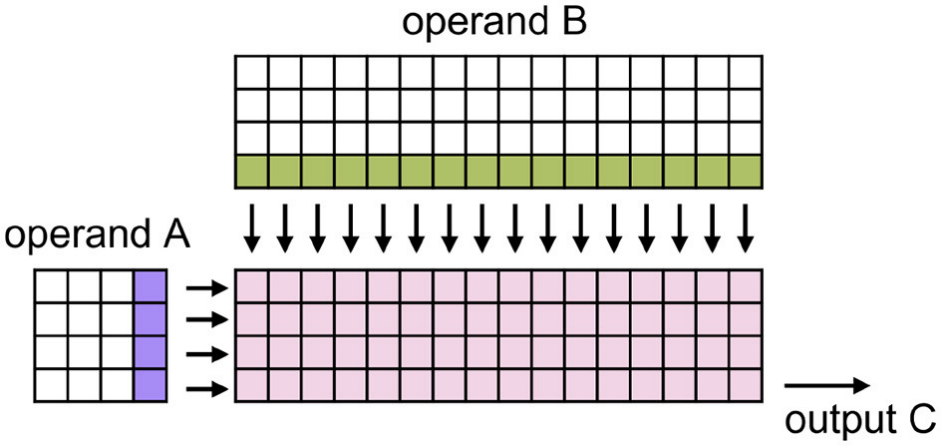
Schematic of the MAC array (Huang et al., 2023). Pink squares express 4 × 16 MAC calculation units. In each clock cycle, the SpiNNaker 2 bus system can convey four values from operand A and 16 from operand B and feed them into the MAC array as the arrow indicates for executing matrix multiplication. To deploy this structure, we should align the row and column number of operand A to a multiple of 4 and operand B to a multiple of 4 and 16.

SpiNNaker 2 is a neuromorphic multi-core system. Each core contains 64 MAC units in a 4 × 16 layout (Yan et al., 2021; Zeinolabedin et al., 2022), which we call the MAC array. For executing matrix multiplication, operands are supposed to be memory aligned, as Figure 1 illustrates. The alignment shapes originate from the fixed hardware architecture of the MAC array and data access bandwidth of the SpiNNaker 2 system. The precision of the operands could be 8 bits or 16 bits. For output precision, 8 bits, 16 bits, and 32 bits can be configured.

### 2.2. Operand Stack

Conducting SNN inference generally consists of two consecutive steps: synaptic processing and neural update. The following equation defines the dynamics of the arguably most prominent neuron model in the brain, that is, the leaky integrate-and-fire (LIF):


(1) $$\begin{array}{l} V_{j}^{t+1}={\text{Σ}}_{i}W_{ji}x_{i}^{t-d\left(j,i\right)}+\alpha V_{j}^{t}-z_{j}^{t}V_{th} \end{array}$$

In the synaptic processing step, weights that connect pre-neuron *i* and post-neuron *j* are summed if the spikes arrive at the post-neuron after traveling across the synapse for time interval *d*(*j, i*). Then, in the neural update step, the membrane potential decays by factor α (equals to $e^{1/\tau_{m}}$, τ_*m*_ denotes the membrane time constant) and is updated by checking the neuron states $z_{j}^{t}$ at time *t*. If the sum of the first two terms exceeds the threshold *V*_*th*_, neuron states are set to 1 and neuron spikes, otherwise 0. This equation refers to (Bellec et al., 2020) and its supplementary, with factor 1−α removed. Unlike the original equation, the input and recurrent synaptic processing share the same term.

**Figure 2:**
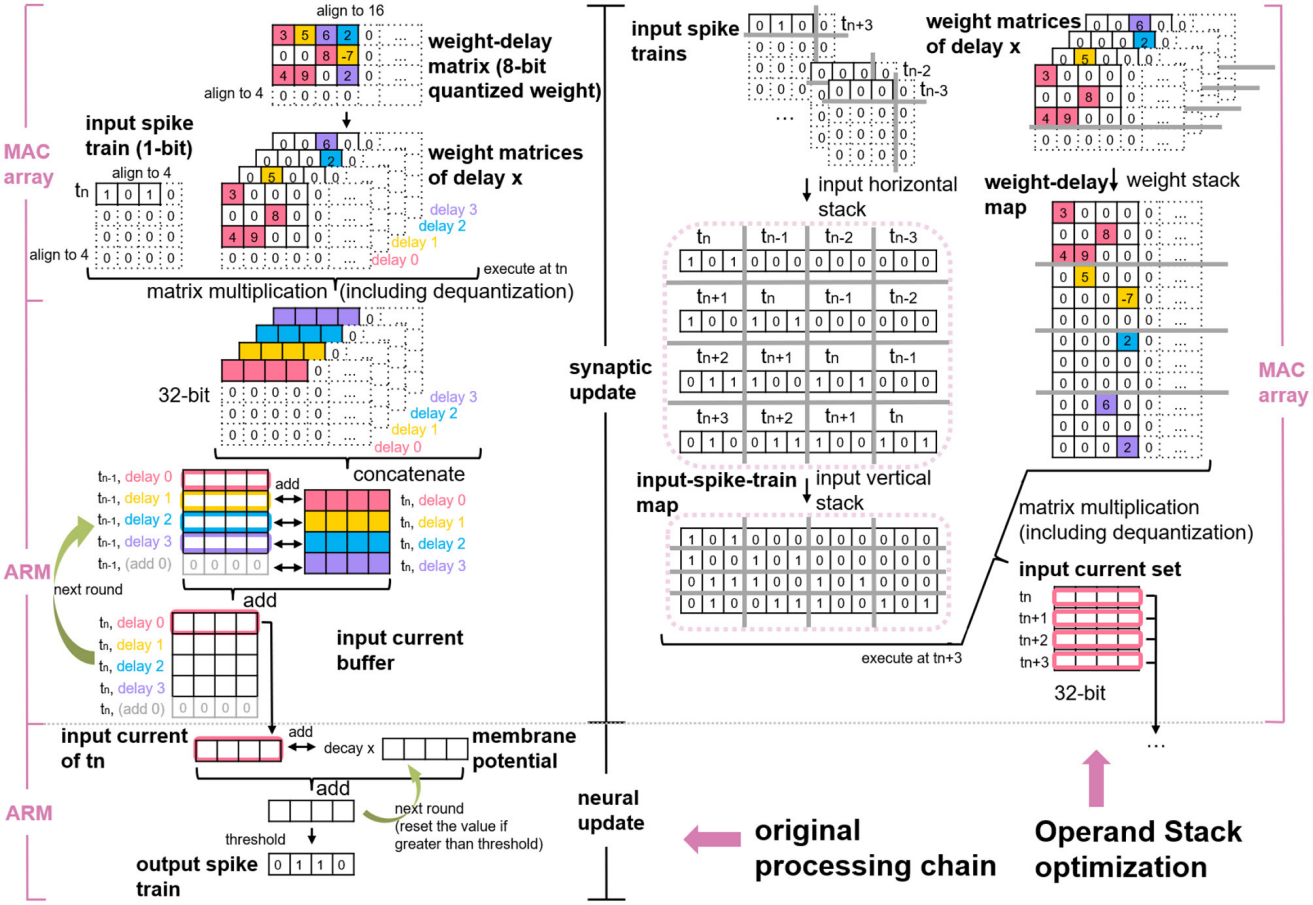
Original processing chain of MAC array calculating SNN **(left)** and the schematic of Operand Stack optimization algorithm **(right)** (Huang et al., 2023).

Because of the high data precision requirement of the neural update, the MAC array primarily contributes to accelerating the synaptic processing of the SNN inference. As with the serial synaptic processing mentioned by Rhodes et al. (2018), the parallel synaptic processing also contains processing input spikes and advancing the input current buffer of each delay, as shown on the left side of Figure 2. To be specific, first, the memory-aligned weight-delay matrix is divided into several weight matrices, each of which has the same delay attribute. Then weight matrices is transmitted to the MAC array one by one serving as the operand B and simultaneously conveying the memory-aligned input spike train to MAC as the operand A. Finally, the MAC calculated results is added to the input current buffer to update the input current of each delay. Here, the “delay,” or “synaptic delay” precisely, is the time for conducting a signal across a synapse, that is, the interval between the arrival of the spike and the start of the membrane potential.

The delay stack algorithm proposed in our previous study (Huang et al., 2023) simplifies these conventional synaptic processing steps to only one step (matrix multiplication), so there is no need to consider the weight matrix division and the input current accumulation. As depicted on the right side of Figure 2, this algorithm stacks sequential input spike trains of *t*_*n*−3_ to *t*_*n*+3_ into an input-spike-train map and stacks weight matrices along the delay into a weight-delay map. These two maps act as the new operands for matrix multiplication on the MAC array. SNN features sparse input to mimic the working mechanism in the mammalian brain but decouples weight sparsity. By applying delay stack, the weight matrix is divided into multiple sparse matrices, and the merged weight-delay map exhibits sparsity as a result. Thus, the issue of accelerating SNN inference with MAC array is converted into efficient multi-core processing SpGEMM problem.

**Figure 3:**
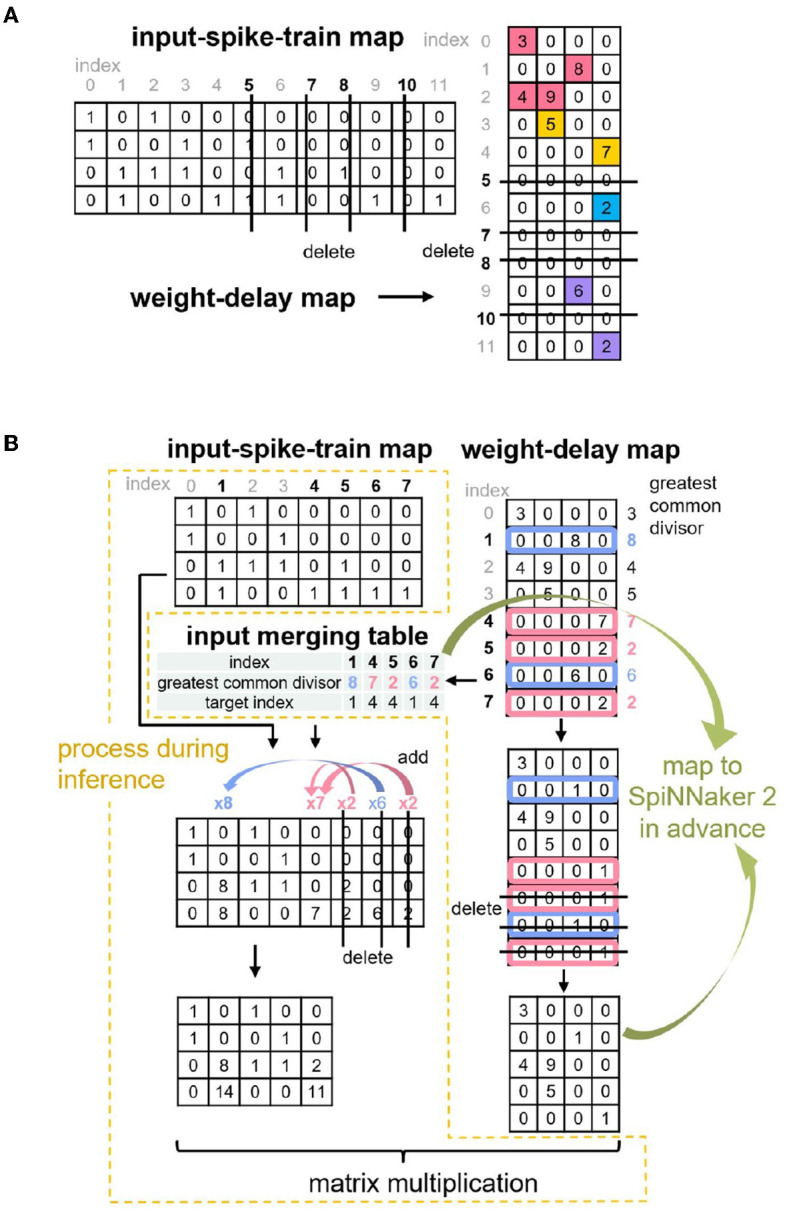
Two optimization algorithms proposed in Huang et al. (2023): **(A)** Zero Elimination **(B)** Proportion Merger.

Considering the limited SRAM space on each PE of SpiNNaker 2 and the sparsity of the merged weight-delay map, our last study (Huang et al., 2023) further applies the Zero Elimination and Proportion Merger algorithms to shrink the size of the matrix-multiplication operands, as shown in Figure 3. Basically, the Zero Elimination algorithm removes the rows with all zero values in the weight-delay map against the operand sparsity, and it also removes the corresponding columns in the input-spike-train map to guarantee the result correctness of the matrix multiplication. The Proportion Merger merges rows that is proportional to each other to only one row for weight-delay map and records the proportional values (greatest common divisor), which contribute to pre-processing the input-spike-train map at runtime. This algorithm essentially migrates some weight-delay information into input operand, addressing the accuracy mismatch problem of SNN input (1-bit) and MAC operand requirement (8-bit/16-bit) and improving the memory utilization.

These three algorithms from our last study (Huang et al., 2023) tackle or alleviate most of the memory issues caused by SNN characters and memory alignment, except memory alignment alongside the column of operands B and C, as indicated in Table 1. In other words, if only deploying these three algorithms, the part from the 5th to 16th column of the weight-delay map in Figure 2 that has all values equal to zero has to be saved on SRAM of PE for matrix-multiplication calculation. According to the MAC array working mechanism mentioned in Section 2.1, the output (operand C) also requires the same number of column reserved for storing matrix-multiplication results, which is unfriendly to the limited memory space. In addition, the previous study does not discuss multi-core MAC arrays collaborating on processing a large SNN model. We address these two issues in the following section.

**Table 1:** Correspondence between memory issues and optimization algorithms.

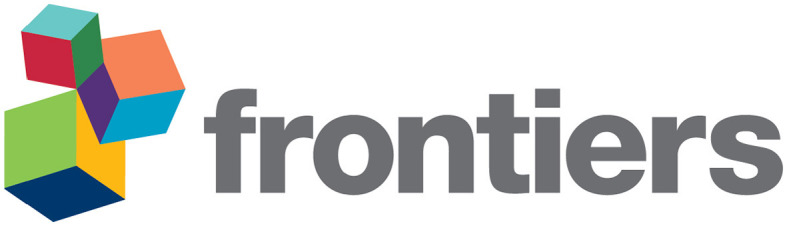 **Memory issuesOptimization algorithms**			**Operand Stack**	**Zero Elimination**	**Proportion Merger**	**Echelon Reorder**
SNN characters	Sparse operand A			⋆	⋆	
	Accuracy mismatch				⋆	
MAC structure						
	Operand A	Row	⋆			
		Column	⋆			
	Operand B	Row	⋆			
		Column				⋆
	operand C	Row	⋆			
		Column				⋆
Generated during	Sparse operand B			⋆	⋆	⋆
optimization						

The first row lists four optimization strategies, and the first and second column state the memory issues to be solved when we want to accelerate SNN inference with MAC array. Stars mark which optimization algorithms can solve or alleviate which memory issues. The first three algorithms proposed in our previous study (Huang et al., 2023) ignore the memory alignment problems of column of operand B and C, highlighted with pink color. The Echelon Reorder algorithm proposed in this study can solve them.

## 3. Method

This section elaborates on each part of the SNN multi-core MAC array acceleration processing chain, incorporating the information densification algorithm for the weight-delay map and input-spike-train map, pure MAC and mixed processor splitting strategies, as well as the multi-core deployment.

### 3.1. Echelon Reorder

#### 3.1.1. Weight-delay map

**Figure 4:**
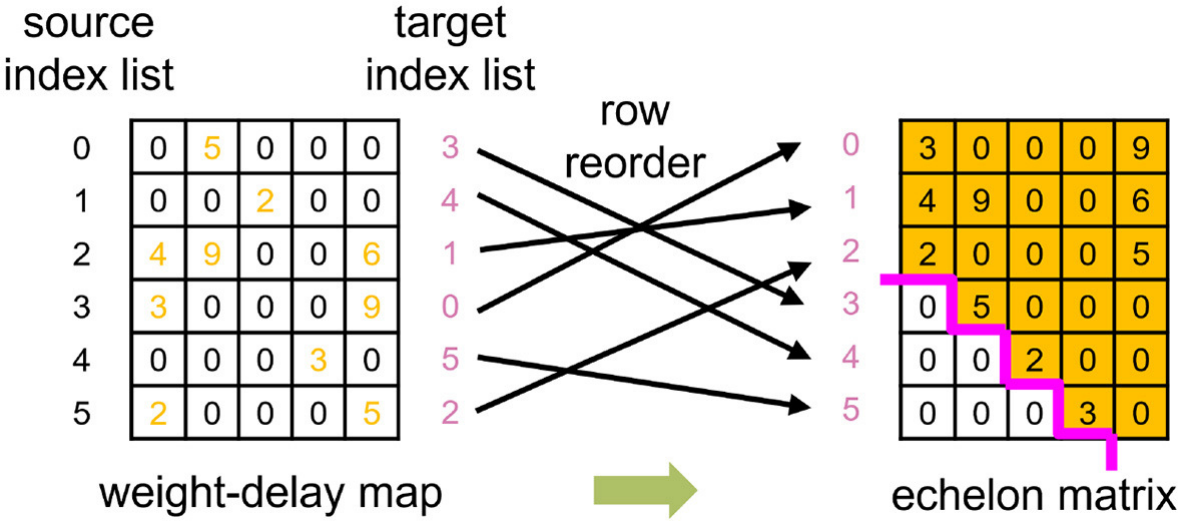
An example of utilizing the Echelon Reorder algorithm, which reorders the row of weight-delay map to form the echelon matrix that the left corner has only zeros.

The Operand Stack algorithm from Huang et al. (2023) dilutes the original weight-delay matrix and results in a sparse weight-delay map. Our proposed Echelon Reorder algorithm takes advantage of this sparsity to isolate the meaningful weights for weight-delay map, as shown in Figure 4 and Algorithm 1. In this algorithm, we reorder the rows of the weight-delay map to form an echelon matrix, of which the lower left part has all values of zero so that the upper right part has a denser distribution of non-zero weights than the weight-delay map. The storage performance and computing power improvement are foreseeable if only the upper right part is stored and calculated. We will discuss the specific storage approach in Sections 3.2 and 3.3.

**Algorithm 1:** Echelon Reorder algorithm.

Require: weight-delay map *WD*_*map* of size *row* × *col*
Ensure: echelon matrix *E*_*matrix*, echelon matrix index list *tgt*_*idx*_*list*
/* this algorithm reorder the rows of *WD*_*map* to generate *E*_*matrix* */
/* execute this algorithm on PC before inference, and load the result *E*_*matrix* to PE of SpiNNaker 2 as operand B */

*E*_*matrix*_*row*_*count*←0
for *j* ← 0 to (*col*−1) **do**
for *i* ← 0 to (*row*−1) **do**
if *WD*_*map*[*i*][*j*]! = 0 **then**
*E*_*matrix*[*E*_*matrix*_*row*_*count*]←*WD*_*map*[*i*][:]
*E*_*matrix*_*row*_*count*+ = 1
add *i* to *tgt*_*idx*_*list*
end **if**
end **for**
end **for**

#### 3.1.2. Input-spike-train map

To guarantee the correctness of the matrix multiplication result, it is necessary to adjust the operand A (input-spike-train map) according to the modification of operand B (weight-delay map) which is discussed in Section 3.1.1. To be specific, we record how the row of operand B is reordered and apply it to the column reorder of the operand A. Unlike the operand B, the operand A is unknown in advance because there is no way to predict the input spike status, so we cannot reorder operand A offline and have to adjust the operand A when executing the SNN inference. This adjustment can be achieved on the host PC with Python or on SpiNNaker 2 with C language. To make a fair comparison with the pure ARM baseline in Section 4 and to extend the capability of SpiNNaker 2 of directly handling the spikes from the sensor peripheral, we propose the Circle Reverse algorithm (Algorithm 2) to online real-time process the input-spike-train map on SpiNNaker 2. It consists of two steps: find circles and reverse circles.

**Algorithm 2:** Circle Reverse algorithm.

Require: weight-delay map index list *src*_*idx*_*list*, echelon matrix index list *tgt*_*idx*_*list*
Ensure: *reversed*_*circle*_*list*
/* this algorithm calculates *reversed*_*circle*_*list* needed for the input-spike-train map online reorder */
/* execute this algorithm on PC before inference, and load the result *reversed*_*circle*_*list* to PE of SpiNNaker 2. It processes input data during inference to generate operand A */

/* step 1: find circles */
/* input: *src*_*idx*_*list*, *tgt*_*idx*_*list* */
/* output: *circles*_*list*, *circle*_*count* */
*circle*_*count*←0
repeat
*start*_*src*_*index*← one element from *src*_*idx*_*list*
*src*_*index*←*start*_*src*_*index*
delete *src*_*index* from *src*_*idx*_*list*
add *src*_*index* to *circles*_*list*[*circle*_*count*]
repeat
*tgt*_*index*←*tgt*_*idx*_*list*[*src*_*index*]
*src*_*index*←*tgt*_*index*
delete *src*_*index* from *src*_*idx*_*list*
add *src*_*index* to *circles*_*list*[*circle*_*count*]
until *tgt*_*index* = =*start*_*src*_*index*
*circle*_*count*+ = 1
until no element in *src*_*idx*_*list*

/* step 2: reverse circles */
/* input: output of step1, that is: *circles*_*list*, *circle*_*count* */
/* output: *reversed*_*circle*_*list* */
for *i*←0 to *circle*_*count* **do**
*reversed*_*circles*_*list*← reverse the elements of *circles*_*list*[*i*]
end **for**

**Figure 5:**
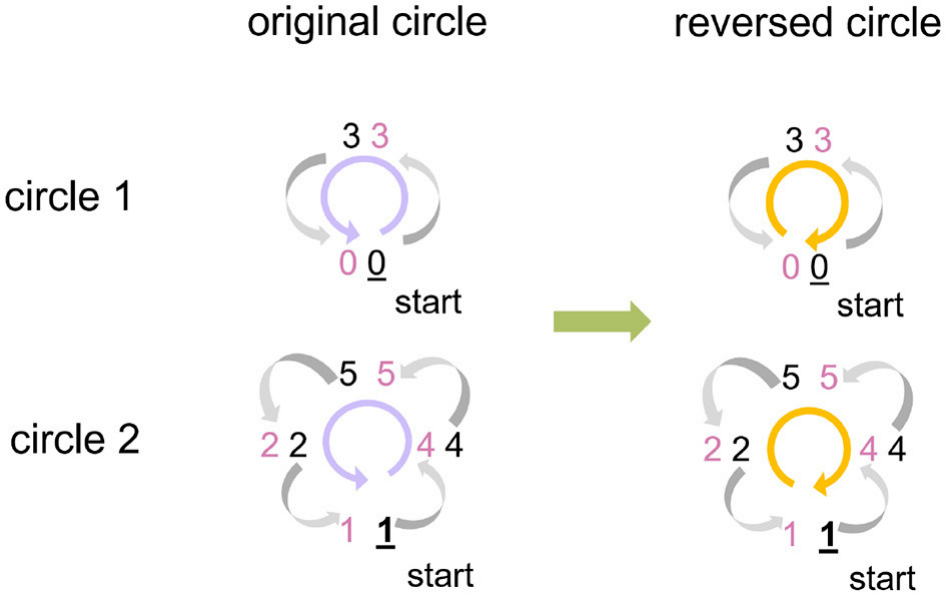
An example of utilizing Circle Reverse algorithm. The indices of the weight-delay map in Figure 4 has two circles with index order 0 → 3 → 0 and 1 → 4 → 5 → 2 → 1, arrows pointing at the direction of the row movement. To avoid the row overwrite (row of weight-delay map, equivalent to column of input-spike-train map here) and save memory footprint, we reverse the index order to: 0← 3← 0 and 1← 2← 5← 4← 1. Preserve the start point of each circle (0 and 1, respectively) beforehand and assign the preserved values to the row that the start row points at (3 and 4, respectively).

Find circles: During the reorder process of the operand B, we get the target index list that corresponds to the source index list, that is, which source row of the operand B should be placed in which target row in the generated echelon matrix, as presented in Figure 4. Suppose the current target index is taken as the next source index, we can retrieve the next target index iteratively until finding out the target index that equals the start source index. The indices found in this process can form a circle. All the indices of a weight-delay map can be represented by several circles. In our example, two circles are found, as demonstrated on the left side of Figure 5.

Reverse circles: Now we consider adjustment of the operand A (input-spike-train map). If we directly move columns of operand A based on the order given by original circles, the previous column overwrites the current column, and then the current column cannot assign the correct values to the next column. To solve this issue, we reverse the index order of each original circle and preserve the start column before executing the column movement for the operand A, as shown on the right side of Figure 5. Then we assign the preserved start column to the column at which the start column points. In this process, only a little extra memory is required to preserve the start column instead of a whole space with the same size as the original input-spike-train map. This algorithm does not involve the sort and search algorithms. Thus, the runtime grows linearly with the number of columns, and the time complexity is O(n).

### 3.2. Multi-core two-stage splitting

#### 3.2.1. Pure MAC

**Figure 6:**
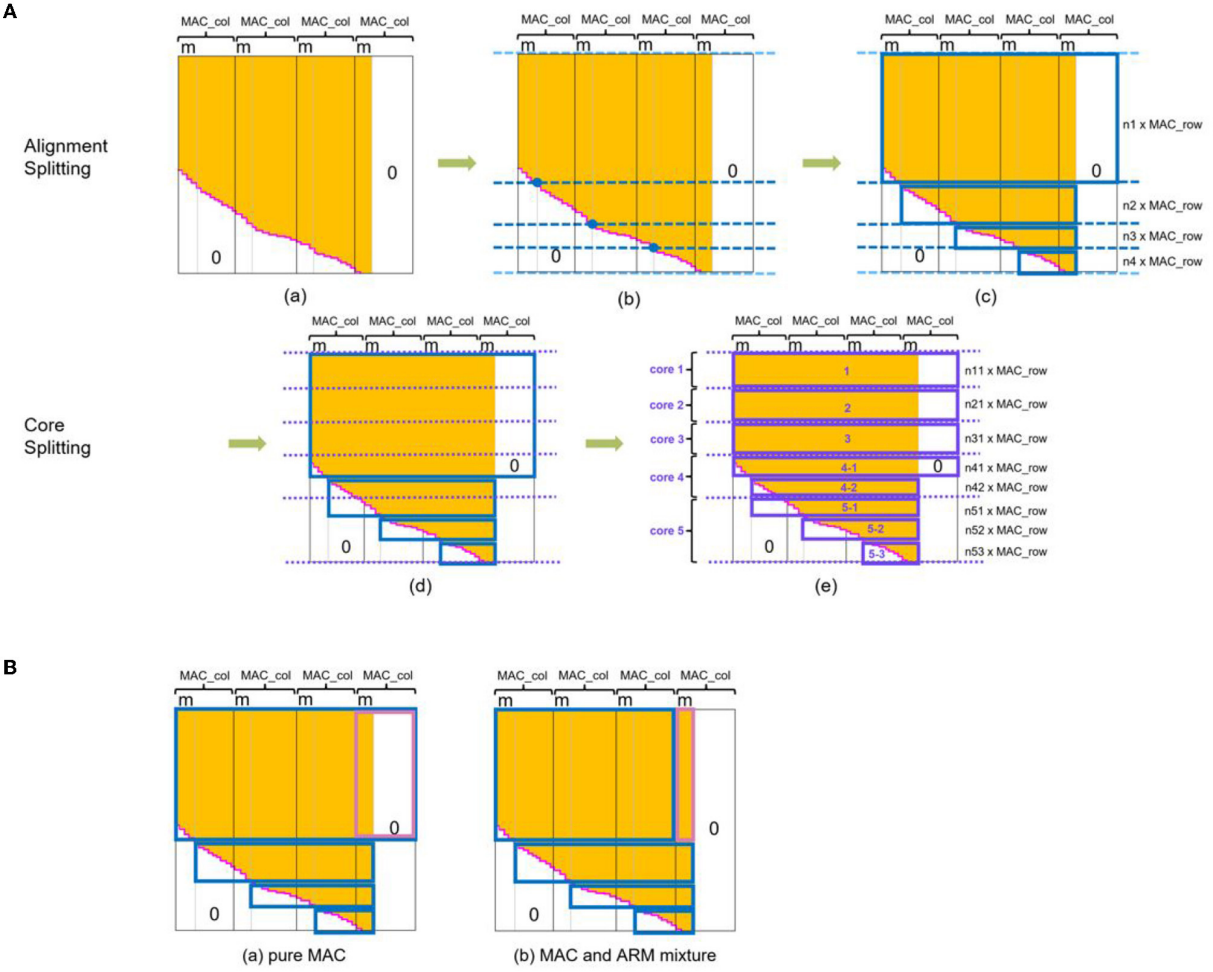
Multi-core splitting. **(A)** Two-stage splitting algorithm. The first splitting stage (alignment splitting) generates a set of rectangles that meet the memory alignment requirement from MAC array hardware structure, i.e., the length of the rectangles is a multiply of MAC array column number (16 for SpiNNaker 2) and the width of MAC array row number (4). The second splitting stage (core splitting) further disassembles and reorganizes these rectangles according to the available storage and then deploy them to multiply cores in a balanced loading way. Algorithm details (a) signifies the memory-aligned echelon matrix. For alignment splitting, first we find the intersection of the gray vertical line and rose red echelon line and determine the dark blue horizontal dividing lines in (b). In addition, add two light blue lines on the first and last rows. Starting from the second line, shift the line downwards until meeting the requirement that the number of rows between two adjacent lines is the multiply of 4. Based on them, we can outline the smallest rectangular set in (c). In the core splitting process, we divide the sum of the memory of all data in the dark blue rectangles by the available memory per PE for getting the number of PEs (supposing 5 in this example). Purple lines in (d) cut the memory into equal five portions at first, and a shift downwards is also necessary if two adjacent lines are not the multiply of 4. Purple rectangles surround the area as (e) depicts. Finally, we can save weights in these rectangles into corresponding PEs for direct MAC calculating with no need for further data format adjustment. **(B)** Two kinds of splitting approaches for echelon matrix. We store the weights enclosed by colored borders into PE. Approach of (a) fits pure MAC acceleration and has the speed advantage. (b) Deploy mixed processors to execute matrix multiplication. The weights outlined in blue utilize MAC array, while the pink area, which is different from (a), uses ARM processor. This approach consumes more running time but less memory footprint. The white area represents zero values, the orange area marks denser weights, and the rose red echelon line is the dividing line of them. *m* represents the remainder of dividing the column number of the echelon matrix by that of MAC array (16). *n* in (c) and (e) of subfigure **(A)** is the non-zero integer.

Considering the MAC array exclusively supporting the acceleration of the rectangular matrix, we employed a set of rectangles to enclose all the meaningful data and as few zeros as possible. The length of the rectangle is a consecutive integer (1, 2, 3, etc.) multiple of 16, and the width is an integer multiple of 4. The multipliers derive from the hardware characteristic of the MAC array of SpiNNaker 2. Using the alignment splitting algorithm, we obtained a set of rectangles with the smallest total area, as Figure 6A(c) indicates. *m* in this figure represents the remainder of dividing the column number of the echelon matrix by that of MAC array (16). The data contained in this set of rectangles consume the least memory resources when deploying on SpiNNaker 2.

Now, length and width of the rectangles meet the requirements of the MAC calculation, and its time to discuss the load balancing issue. The amount of data outlined by rectangles varies. All data in some rectangles might occupy little memory if we simply distribute each small rectangle to one PE without combination. In contrast, all data in other rectangles may be far beyond the maximum available SRAM space of one PE. Even if the data in the largest rectangle barely fit into one PE, the unbalanced weight loading of the rectangle set among multiple PEs can prolong the overall processing time, and partial computing power of the core with a low weight load is wasted. Therefore, based on the set of rectangles obtained in the above steps, we execute the core splitting algorithm to achieve a spatial-temporal load balancing deployment on multiple cores. This step splits the rectangles obtained by alignment splitting into more and smaller rectangles horizontally and then divides them into several groups with an equal amount of weight values, as Figure 6A(e) shows. The number of the group is the number of PEs to be activated.

Equally distributing weights across multiple cores has many possibilities. Here, we put weights into as few PEs as possible to fully utilize the resources of each core. We do this for two reasons: on the one hand, matrix multiplication with MAC array has a considerable time advantage, and the marginal utility of dividing into more cores is tiny; on the other hand, the full utilization of each core is also conducive to simultaneously deploying and executing more tasks on multi-core SpiNNaker 2 platform in future.

#### 3.2.2. MAC and ARM mixture

By observing the biggest rectangle in Figure 6A(c), we find that there is still a relatively large area with all values equal to zero, arising from memory alignment alongside the column of operand B. The concrete size of this area is subject to *m*, the remainder dividing the column number of the echelon matrix by 16. When *m* is small, these zero values employed as placeholders occupy a large memory space. We improve this situation by abstracting only the meaningful data, as Figure 6B(b) illustrates, and conducting matrix multiplication for this part with the ARM core.

As for the corresponding core splitting strategy that matches this mixture alignment splitting, we figured out the number of required PEs with the approach mentioned above and outlined the core splitting rectangles for the blue and pink marked area of Figure 6B separately. For example, we needed four PEs, so we split the whole area marked with blue into four groups equally and do the same for the pink. Later, in the deployment step, we saved one split rectangular group for MAC calculation and one for ARM into one PE. Then all the activated PEs executed matrix multiplication by leveraging the local MAC array and ARM core. Finally, the results from all activated PEs were converged to get the synaptic processing result.

This optimization eliminates the extra memory overhead originating from necessary memory alignment alongside the columns of operands B and C. When *m* is small, or the number of source neurons (corresponds to the row number of echelon matrix) is large, the optimization is particularly effective.

#### 3.2.3. Pure or mixed?

**Figure 7:**
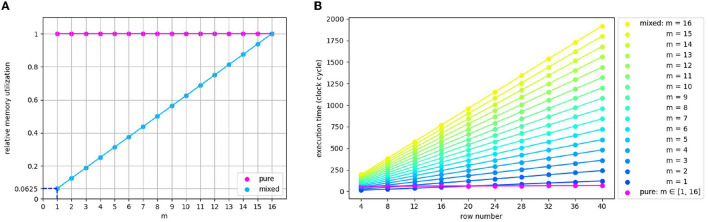
**(A)** The memory usage comparison of pure and mixed processor approaches. **(B)** Synaptic processing execution time comparison of pure MAC array and ARM part of the mixed approach, regarding the part that has difference when adopting the pure or mixed approach. Pink rectangles in Figure 6B mark this part. *m* represents the remainder of dividing the column number of the echelon matrix by that of MAC array (16).

The variable *m* represents a number ranging from 1 to 16 in various models. Therefore, it is necessary to analyze the influence of *m* on processor selection quantitatively. In the pink outlined area in Figure 6B(a), the memory cost of the pure MAC approach is independent of m. In contrast, the MAC and ARM mixture approach consumes only 6.25% of the memory required by pure MAC in the extreme case of *m* being 1, as shown in Figure 7A. With *m* climbs, the memory gap gradually narrows until it disappears when *m* reaches the maximum value of 16. As for the time comparison, we found that pure MAC outperforms ARM of the mixed approach except for several cases when *m* and row number are pretty small, according to Figure 7B. Consequently, if a model has a high requirement of the real-time reaction and fewer memory constraints, pure MAC is a better option in the vast majority of cases; otherwise, MAC and ARM mixture outperforms.

### 3.3. Multi-core authorization deployment

**Figure 8:**
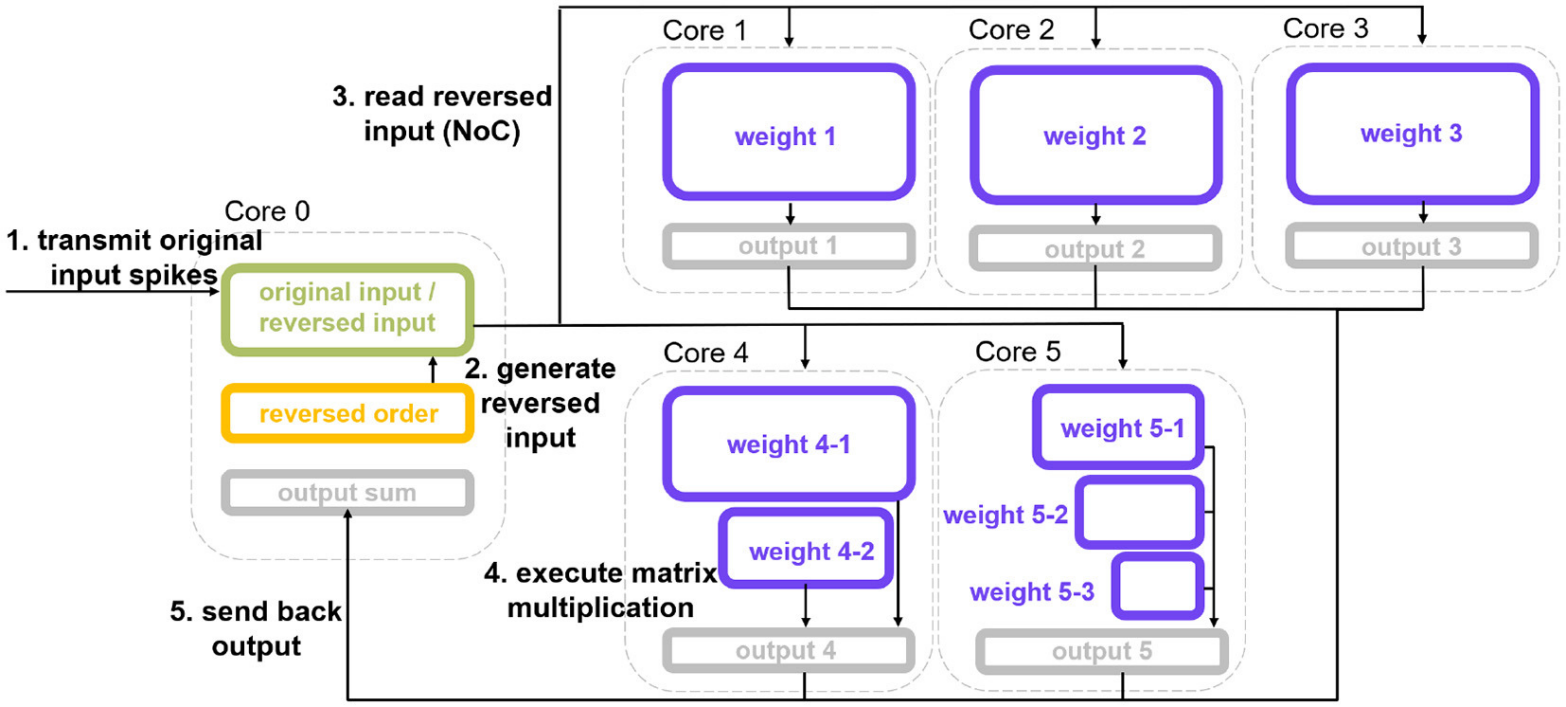
Multi-core authorization result and SNN multi-core MAC array acceleration processing chain. Core 0 is the ”Dominant PE,” and Core 1 to Core 5 are “Subordinate PEs.” The weights deployment corresponds to the splitting outcome of Figure 6A, i.e., pure MAC splitting. This processing chain is also applicable to the outcome of MAC and ARM mixture splitting.

After the splitting process, we discuss how to deploy the split echelon matrix and where to process the original input-spike-train map. The MAC array of SpiNNaker 2 supports reading operands from other PEs. Based on this feature, we authorize the core that reads reversed input data from another PE and preloads the split weight rectangular group as the “Subordinate PE.” The PE that provides reversed input data acts as the “Dominant PE.” Figure 8 illustrates the multi-core authorization result and the whole processing chain. First, the Dominant PE (Core 0) receives the original input spikes, generates the reversed input-spike-train map, and waits for the reading request from Subordinate PEs. Then, the Subordinate PE reads the corresponding reversed input data and performs pure MAC operation or mixture calculation. The generated synaptic processing results are eventually accumulated and written back to the Dominant PE, serving as the input for the subsequent neural update step.

## 4. Experiment and result

To evaluate the performance of the information densification and splitting algorithms and the feasibility of the deployment strategy from Section 3, we benchmarked it with constructed and actual SNN models in this section.

### 4.1. Constructed SNN models

The optimization performance of our proposed approaches relies on the following five factors: delay range, number of pre-neurons, number of post-neurons, weight connection density, and the selection of splitting strategy. The first four factors are determined by the SNN model itself. Thus, we constructed multiple SNN models based on various combinations of these four factors and explored their influence on spatial and temporal performance. To focus on the scope of our proposed approaches, we experimented on the synaptic processing part of SNN.

**Figure 9:**
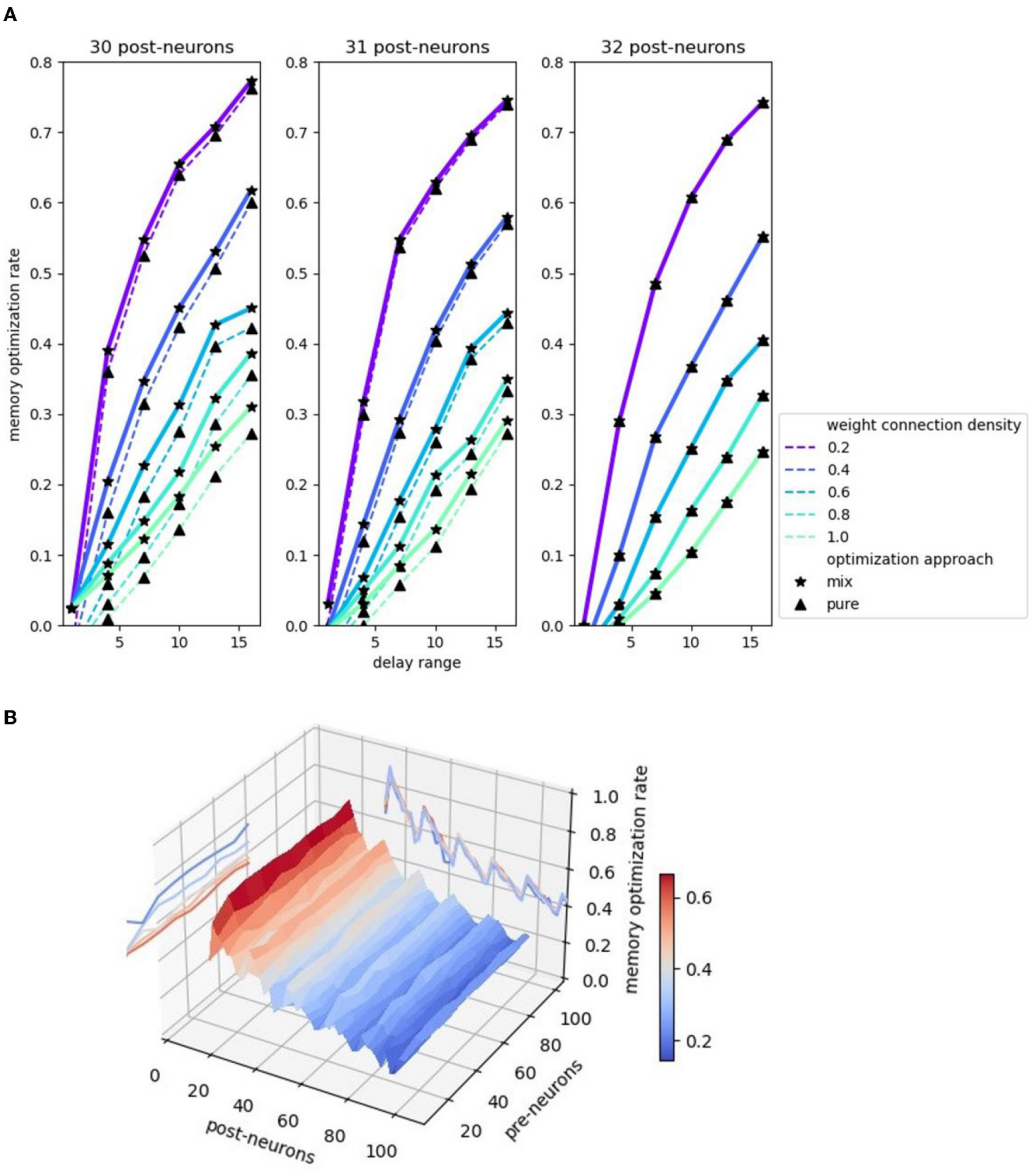
Memory optimization rate comparison. **(A)** Memory optimization rate comparison 2D plot. Three plots corresponds to 30, 31, 32 post-neurons. Pre-neuron is set to 50. Delay ranges from 1 to 16. **(B)** Memory optimization rate comparison 3D plot with the projections of the contours.

Figure 9 illustrates the comparison of the memory optimization rate among 30, 31, and 32 post-neurons. We define memory optimization rate as the ratio of the number of uncalculated weights to the number of memory aligned weights, i.e., the ratio of the area that is not enclosed by rectangles to the total area in Figure 6B. Figure 9A shows that the memory optimization effect gets better with the increase of delay range. Moreover, the two echelon algorithms perform better in memory cost when the weight operand is getting more sparse, and the more sparse the weight operand, the more obvious the effect. In addition, the mixed splitting approach always performs better than or equal to the pure MAC approach. This experimental result is consistent with the analysis in Section 3.2.3. The performance difference between these two splitting approaches depends on the remainder of post-neurons divided by 16, that is, the value *m* in Figure 6. As the remainder grows, the performance difference decreases. Until the remainder reaches 16 (back to 0), there is no difference between the two methods.

In addition to the impact of the remainder of post-neurons divided by 16 on the spatial performance difference between the two methods, the change of the post-neuron number itself also affects the memory optimization rate. Figure 9B demonstrates a 3D plot with the projections of the contours, highlighting the relation of memory optimization rate and two factors: the number of pre-neurons and post-neurons. Observing the contour on the back “wall,” we find that the memory optimization rate fluctuating declines with a period of 16 with the post-neurons increasing. The increase of post-neurons brings the reduction of the optimization rate, which surges at the point where the remainder returns from 15 to 0. The reason is that the post-neuron number at this point exactly adapts to the hardware structure of the MAC array that no memory alignment is required. The projected contour on the left “wall” is almost parallel to the pre-neurons axis after the initial unstable status, implying the independence of pre-neuron number and memory optimization rate. The following formula briefly summarizes the relationship between memory optimization rate and factors:


(2) $$\begin{array}{l} r_{opt}\;\underset{\sim }{\text{∝}}\;d,\frac{1}{p},\frac{1}{n_{post}} \end{array}$$

*r*_*opt*_ represents the memory optimization rate, which is approximately directly proportional to the delay range *d*, and inversely to weight connection density *p* and number of post-neurons *n*_*post*_. The use of the approximately proportional symbol $\underset{\sim }{\text{∝}}$ is intended to qualitatively show the positive and negative relationship of the variables before and after it. The relation details, such as the aforementioned “the more sparse the weight operand, the more obvious the effect” and “fluctuating declines with a period of 16,” are not reflected in this equation.

Although the memory optimization rate is unrestricted by the pre-neuron number, the impact of the pre-neuron number on SNN inference performance is mainly reflected in the running time, which basically consists of input data pre-processing (Circle Reverse algorithm elaborated in Section 3.1.2) and synaptic processing. The synaptic processing is accelerated by the MAC array, and the input data pre-processing is only calculated by ARM. Therefore, the input data pre-processing consumes most of the temporal resources. The smaller the number of input neurons (i.e., pre-neurons), the better the algorithms proposed in this study performs in execution time. If only considering the synaptic processing part, the temporal performance of the mixed echelon approach also has an intense dependence on the number of pre-neurons. The mixed echelon algorithm sacrifices some runtime in exchange for a lower memory footprint, and this time is positively correlated with the number of pre-neurons.

### 4.2. Actual SNN models

We selected two actual benchmarks for evaluation of our proposed approaches: an application model from the real scenario (radar gesture recognition SNN model) and a classic structural modeling in neuroscience (balanced random cortex-like network). The experiments compared serial ARM, original MAC, pure MAC echelon, and MAC and ARM mixture approaches regarding spatial-temporal performance. The data measurement concentrates on the part where the above four approaches behave differently during SNN inference, that is, synaptic processing. As for the multi-core mapping and deployment, we evenly split the weight operand into multiple adjacent PEs and fully utilized each PE's available memory resources (120 KB in our configuration) in conjunction with other necessary data (split input, temporary output, and input current buffer) for serial ARM and original MAC. When a PE can accommodate not less than one weight matrix of one delay, we ensure the integrity of a computational unit, i.e., one weight matrix of one delay, to avoid introducing additional result fusion time. For example, if a kernel can hold 2.5 weight matrices of one delay, we assigned pure MAC echelon and mixture approaches that adopted supporting strategies from Section 3.2 and 3.3.

We adopted the following method to calculate memory cost and measure the execution time of the multi-core cooperation system:

- Two baselines: The memory cost of serial ARM and original MAC comes from the input placeholder, weight, output placeholder, and input current buffer in all activated PEs. Note that the input and weight operands are supposed to be memory aligned in advance. The execution time is comprised of matrix multiplication, output merging from different PEs and input current buffer update.
- Two echelon approaches: The memory cost consists of the footprint of the Dominant PE (input placeholder, reversed order, and output) and Subordinate PEs (weight cost and temporary output). In addition to the matrix-multiplication execution time (MAC or mixture processors) and the time of accumulating output into Dominant PE, the execution time also incorporates the input reorder time to guarantee a fair comparison with serial ARM and the original MAC baseline. Since each calculation obtains the same number of results as the delay number instead of only one result of the serial ARM and original MAC approach as Figure 2 shows, we divide the tested total execution time by the delay number to get the processing time per frame.

#### 4.2.1. Application model from the real scenario: radar gesture recognition SNN model

**Figure 10:**
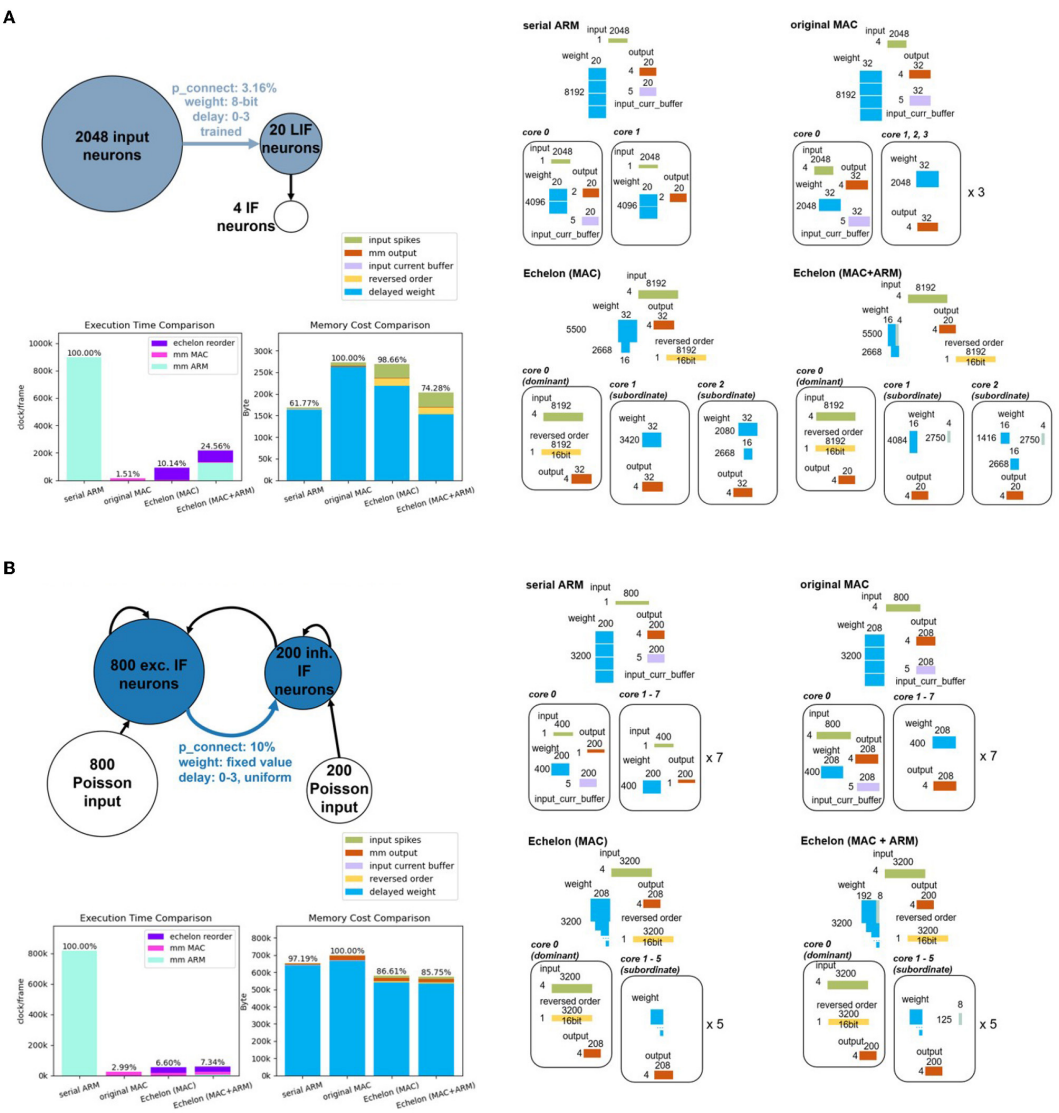
**(A)** Top-left is the structure of the radar gesture recognition model. The experiment part corresponds the synaptic processing of the blue-marked components, including the neural populations and the synaptic projection. The right subfigures depict what experimental memory objects are required and how they are split and deployed into multiple PEs of SpiNNaker 2 hardware according to four different synaptic processing approaches (two baselines and two proposed approaches). The two bar plots on the bottom left compares the execution time and memory cost of these four approaches. The colorful legend in the middle fits the memory cost comparison bar plot and all the subfigures on the right. **(B)** Balanced random cortex-like network has the same subfigure distribution as **(A)**. Because of the limited space, we put the specific splitting result of two echelon algorithms of the balanced random cortex-like network into Supplementary material.

Gesture recognition is an important and active area of AI research, with relevant models and hardware deployment acting as the fundamental verification unit for more sophisticated real-world scenarios. For the first benchmark, we set up the radar-based SNN gesture recognition model with 2,048 input neurons, 20 hidden neurons, four output neurons, and four delays. This model is similar to the study in Gerhards et al. (2022) and Huang et al. (2022a,b) but introduces the concept of delay. We train this benchmarking model with our own collected radar dataset mentioned in Kreutz et al. (2021), involving three directional gestures (left, right, and push) and one environmental reference (random gesture or background noise). The experiment refers to processing synapses between the input layer and hidden layer marked with light blue in the gesture recognition model on the left top of Figure 10.

For the Echelon Reorder optimization algorithm with only MAC as the processor, the densified weight-delay map (i.e., echelon matrix) can be split and deployed into two Subordinate PEs as depicted on the right part of Figure 10A. One Dominant PE has enough space to save the input-spike-train map and the reversed order. The mixed echelon algorithm also consumes two Subordinate PEs and one Dominant PE. Both serial ARM and original MAC baselines need more than one PE, given the available memory of one PE. We split the weight and input operands of these two baselines into multiple cores in a balanced and compact way for a fair comparison purpose. According to the operand scale, two and four PEs are required for these two baselines, respectively.

Analyzing the performance data reported on the bottom left of Figure 10A, the echelon optimization with mixed processors occupies 74.28% of the memory of the original MAC calculation. It is close to the serial ARM memory cost. As for execution time, it accounts for 24.56% of the serial ARM calculation, i.e., this approach optimizes 75.44% of the runtime. The echelon optimization with MAC even increases this percentage to 89.86% (1−10.14%). The remainder *m* and row number are calculated to be 4 and 5,500. According to the analysis in Section 3.2.3, the mixed optimization is expected to outperform the pure MAC regarding memory footprint but be inferior concerning execution time. The experiment data from Figure 10 are consistent with this deduction.

#### 4.2.2. Classic structural modeling in neuroscience: the balanced random cortex-like network

The balanced random cortex-like network, commonly used to benchmark and map to neuromorphic systems (Brüderle et al., 2010; Pfeil et al., 2013; PyNN, 2023), serves as our second benchmark, originated from Brunel's work (Brunel, 2000). Brunel devoted to models of networks with simple neurons to describe the dynamical properties of sparsely connected excitatory and inhibitory integrated-and-fire (IF) neurons. His study reports that the networks present rich states according to external stimulation, the proportion of excitation and inhibition, and synaptic features.

Similar to the cortex in the brain, the balanced random cortex-like networks are composed of an excitatory and inhibitory population of neurons (PyNN, 2023), which are randomly interconnected with each other with fixed probability and recurrently connected within the population. They are also stimulated with excitatory populations with spikes of Poisson distribution, as shown on the top left of Figure 10B. We choose the main part of the model, namely, the excitatory population projecting the inhibitory population marked with dark blue in the Figure to benchmark the proposed approaches in this study. Compared with the original experiment setup in Brunel (2000), the overall number of neurons is scaled to 1,000, but the proportion of excitation and inhibition remains the same (4:1), i.e., 800 excitatory and 200 inhibitory neurons. The parameter delay keeps the original value four and is uniformly distributed in the range of 0 to 3. We create the connection between excitatory and inhibitory populations with a fixed probability of 0.1.

The pure MAC echelon and mixed echelon require one Dominant PE and five Subordinate PEs according to the splitting and deployment strategies mentioned in Section 3.2 and 3.3. Both serial ARM and original MAC run on eight PEs. The right part of Figure 10B presents the specific splitting and deployment consequences.

The sub-graph in the lower left of Figure 10 compares the spatial-temporal performance of four approaches. Two echelon optimization occupies ~86% of the memory of the original MAC calculation, and both outperform serial ARM. In addition, the execution time of two echelon algorithms is smaller than 7.4% and close to the original MAC.

#### 4.2.3. Performance comparison of the two actual models

The two benchmark actual models coming from different domains have distinct structures that are suitable for comparison. The radar gesture model features a larger number of pre-neuron (2,048) than the balanced random cortex-like network (800), as shown in Figure 10. According to the analysis in Section 4.1, the number of pre-neurons mainly influences the execution time performance dominated by ARM processing parts (Echelon Reorder and ARM calculating part of matrix-multiplication of mixed echelon). Thus, the violet and light blue-marked areas of two echelon algorithms on the execution time comparison subfigure of Figure 10A is larger than that of Figure 10B. The balanced random cortex-like network with a smaller number of pre-neuron has a better spatial optimization result by leveraging echelon algorithms.

In addition to the distinction in pre-neuron number, these two benchmark models structure different weight connection densities (3.16%, 10%) and post-neuron numbers (20, 200). The formula 2 indicates the model with smaller weight connection densities, and post-neuron numbers deserve a better memory optimization effect for weight-delay map. The blue bars of two echelon algorithms relative to the original MAC in the memory cost comparison of Figure 10A are indeed significantly smaller than that of Figure 10B. However, the overall advantage of spatial performance improvement of the radar gesture model is blocked by extra memory for storing input spikes (green bars) and reversed order (yellow bars) owing to a larger number of pre-neurons.

In addition, we introduce a definition of “memory alignment rate of column” to account for the reason for a sharper drop of the blue bar from echelon (MAC) to echelon (MAC + ARM). The memory alignment rate of the column presents the impact of memory alignment along the column of the weight-delay map for the size of the whole memory aligned weight-delay map, that is, the rate of the right side white rectangle area to the whole area in Figure 6A(a) or


(3) $$\begin{array}{l} r_{align\text{\_}c}=\frac{16-m}{n_{post}+16-m} \end{array}$$

where *m* is the reminder of post-neuron number *n*_*post*_ divided by 16. With the increase of the post-neuron number, the memory alignment rate decreases. This means that the extra area brought by column memory alignment is a decreasing share of the total weight-delay map. As a result, the echelon (MAC + ARM) algorithm, functioning as optimizing this extra area as mentioned in Section 3.2.2 and 3.2.3, behaves much better in the radar gesture recognition model than in the balanced random cortex-like network. Similarly, the different ratios (we name the ratio *r*_*align*_*rc*_) in comparison of serial ARM and original MAC (61.77%, 97.19%) are caused by memory alignment along the row and column of weight matrices of delay x shown in Figure 2. A larger post-neuron number leads to a smaller memory increase of memory alignment from serial ARM to original MAC.

## 5. Conclusion and discussion

### 5.1. Summary

This study describes the processing chain for accelerating SNN inference with multi-core MAC arrays, including Echelon Reorder information densification algorithm, Multi-core two-stage splitting and multi-core authorization deployment strategies. These algorithms and strategies alleviate the intrinsic memory issue of excessive usage originating from memory alignment and data sparsity. They also realize the multi-core spatial-temporal load balancing for the large SNN layer. We benchmark with constructed and actual SNN models. The former explores how model feature and algorithm selection affect the spatial-temporal optimization performance, and the latter demonstrates two actual SNN models (the radar gesture recognition SNN model and balanced random cortex-like network) on SpiNNaker 2 hardware. They prove the feasibility of the whole optimization process and achieves performance increase. Based on the theoretical analysis and the experiment result, we found those as follows:

- The proposed algorithms and strategies are applicable to various densities of matrix multiplication operands, and the memory optimization degree increases with the weight operand getting sparse.
- In addition to the weight sparsity, the memory optimization rate is also positively correlated with delay range.
- The number of post-neurons periodically affects the memory optimization rate, and the overall trend is downward.
- The number of pre-neuron is generally independent of the memory optimization performance but has intense correlation with running time.
- The echelon mixed processor algorithm behaves better regarding memory but has less temporal efficiency than the echelon pure MAC solution.

The proposed algorithms not only provide a concrete solution for accelerating SNN on the multi-core MAC arrays of SpiNNaker 2 but also has a referential value for hardware systems embedded with multi-core MAC arrays that intend to solve the SpGEMM issue.

### 5.2. Related work

Some researchers have introduced the parallel computing concept into SNN inference to tackle the problems caused by CPU-based parallel processing. One of the representative works of parallel processors accelerating SNN computation is GeNN (Yavuz et al., 2016), a code generation framework speeding up the SNN simulation process using the graphics processing unit (GPU). Specifically, it speeds up the synaptic processing by utilizing a serially executed atomic add operation to add weight to the delay ring-buffer after reading the index of post-neuron and weight and delay by each thread of the GPU (Yavuz et al., 2016; Knight and Nowotny, 2018). However, this mixture of thread/vector parallel processing and serial add operation does not take full advantage of the parallelism of the processor.

Another related study regarding parallel processing SNN inference is SpiNeMap (Balaji et al., 2020) and its follow-up (Balaji et al., 2021). SpiNeMap is a design methodology to partition and deploy SNNs to the crossbar-based neuromorphic hardware DYNAP-SE (Moradi et al., 2018). The proposed unrolling techniques decompose a neuron function with many presynaptic connections into a sequence of computation nodes with two presynaptic connections. This approach improves the crossbar utilization but introduces spike latency, i.e., distorts interspike intervals (ISIs) for global synapses. This issue can be relieved by reducing the number of spikes on global synapses as reported in Balaji et al. (2020), probably realized by modifying the model parameters or decreasing the input spike rate, both of which can negatively impact the accuracy of the original SNN.

Our study targets no ISIs, no accuracy loss, and a wholly parallel calculation based on the more efficient matrix parallelism rather than vector-based computing.

### 5.3. Macro significance

The proposed algorithms act on synaptic processing, which is part of the SNN inference. The impact of algorithms on the optimization of the whole network is a question worth discussing. To review the motivation of our study, it is discussed in Section 1 that the serial ARM baseline suffers from a large time-consuming issue and the naive parallel solution original MAC is unfriendly to limited memory space, it is necessary to explore the portion of synaptic processing of serial ARM in time and original MAC in memory in order to have a macro view of the degree of optimization of our proposed algorithms in the whole SNN inference.

SNN inference consists of synaptic processing and neural update. We estimated the time consumption rate of these two steps for the serial ARM baseline with the following equation referring to ARM Cortex-M4 Technical Reference Manual (ARM, 2023):


(4) $$\begin{array}{l} \begin{array}{c} \frac{t_{synap}}{t_{neural}}=\frac{n_{pre}\times n_{post}\times d\times MLA}{n_{post}\times \left(MLA+COMP\right)}≊0.67\times n_{pre}\times d. \end{array} \end{array}$$

This equation shows that the time consumption rate depends on the number of pre-neuron and delay range. A meaningful model is supposed to have more than one pre-neurons, so this rate is always greater than 1. For the two benchmark actual models in Section 4.2, the synaptic processing time is 5,488.64 and 2,144.0 times larger than neural update.

As for the memory comparison, suppose that the number of pre- and post-neuron is memory aligned, the decay and threshold values of the neural update of all neurons are identical, and only count one output matrix for the multi-core scenario, we have the following equations:


(5) $$\begin{array}{l} \frac{mem_{synap}}{mem_{neural}}=\frac{4\times n_{pre}+n_{pre}\times n_{post}\times d+4\times n_{post}+4\times n_{post}}{4\times n_{post}+0.125\times n_{post}}\\ \;≊1.94+0.24\times n_{pre}\times \left(\frac{4}{n_{post}}+d\right). \end{array}$$

This equation provides the rate of memory cost of synaptic processing to neural update for the original MAC baseline. The rate always greater than 1.94 means synaptic processing dominates memory cost. For the two benchmark actual models, this rate indicates the memory cost of synaptic processing is 2,066.32 and 773.78 times larger than neural update, respectively.

The above search confirms that synaptic processing has the lion's share in SNN inference, both in terms of running time and memory storage. Therefore, the optimization of synaptic processing is crucial and largely determines the optimization performance of the whole SNN inference.

### 5.4. Limitation and future work

By analyzing formula 2 and comparing two actual models regarding the temporal performance of synaptic processing, we found that the larger number of pre-neuron hinders the running time optimization degree that is primarily governed by ARM calculation parts of the Echelon Reorder and matrix-multiplication of the echelon mixed processor approach. Future optimization for time can be placed on finding a more efficient algorithm for the Echelon Reorder and further compressing the data of the serial operation range in the echelon mixed processor approach.

**Figure 11:**
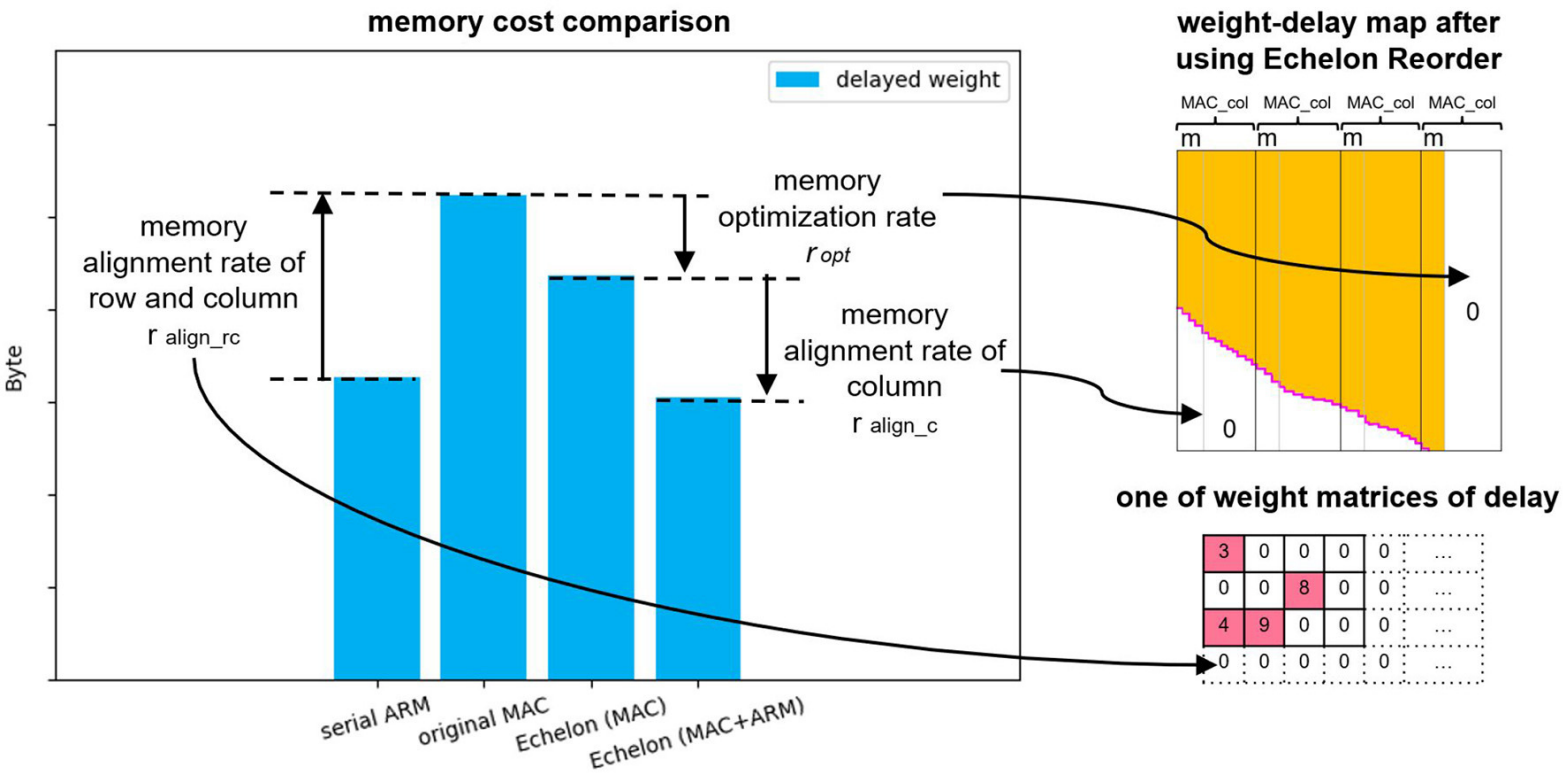
Summary of the memory cost differences of delayed weight of the four approaches (two baselines and two proposed approaches) with three “rate.” The memory alignment rate of row and column *r*_*align*_*rc*_ equals the memory aligned area in the weight matrices of delay x from Figure 2, and other two “rate” represent the proportions of the pointed white area in the whole area of weight-delay map after using the Echelon Reorder.

The spatial performance of our proposed optimization algorithms is limited by the smaller delay range, denser weight connection, and the larger number of post-neuron. How to further optimize the memory optimization rate *r*_*opt*_ and *r*_*align*_*c*_ is defined in Section 4.1 and Section 4.2.3 and increasing the area of the white area of Figure 11 are the next things that are worth investigating. This study merely elaborates on the optimization mechanism of the Echelon Reorder that is based on the data transformation of the Operand Stack. In fact, the other two optimization algorithms (Zero Elimination and Proportion Merger) in Table 1 proposed in our previous study (Huang et al., 2023) of one PE accelerating SNN inference also fit multiply PEs. The memory optimization will benefit from their synergy.

Although Section 3.3 provides the solution of authorizing PEs that contain the split echelon matrix and input buffer, an efficient multi-core deployment strategy and routing algorithm are not included in this study. Randomly deploying the six cores from Figure 8 on SpiNNaker 2 may cause a relatively low-efficient communication between the “Dominant PE” and the “Subordinate PE.” This issue will be of greater concern when we extend the deployment of one SNN layer to an entire network. At that time, a reasonable multi-layer deployment topology and global routing algorithm can avoid the potential traffic congestion and reduce the communication latency by fully utilizing the bandwidth resources of PE to PE and PE to DRAM. Thus, improving the spatio-temporal efficiency of the entire SNN even multiple SNNs on SpiNNaker 2 will be one of our future research priorities.

The traditional serial processing for SNN inference, as the current mainstream method, is constantly being optimized and iterated, and the performance has been improving. It has a good performance in the condition of very sparse input spike train and weight-delay operand, which is what the approaches proposed in this study is yet to be improved. If we can find the sweet spot of SNN model structure factors including input and weight connection density between the traditional approach and our algorithms, it will help the neuromorphic community to have a deeper understanding of the serial and parallel processing methods and contribute to the mechanism of the hybrid processors jointly processing SNN inference in a more efficient way.

## Data availability statement

The data analyzed in this study is subject to the following licenses/restrictions: the data that support the findings of this study are available from the corresponding author upon reasonable request. Requests to access these datasets should be directed at: Jiaxin.Huang@infineon.com.

## Author contributions

JH, CL, and CM conceived the study. JH implemented the optimization system with Python (supported by FKr and PG), carried out the experiment of constructed SNN models (supported by DS), and performed the actual SNN models mapping and emulating on SpiNNaker 2, based on the low-level hardware design and communication library from FKe. BV helped JH with the basic structure of SNN middle-ware development. KK and CM supervised the findings of this study. All authors discussed the results and contributed to the final manuscript.
