# Supplementary material for: Efficient SNN multi-cores MAC array acceleration on SpiNNaker 2
Source: Front Neurosci. 2023 Aug 7;17:1223262. doi: 10.3389/fnins.2023.1223262 (PMC10440698; doi:10.3389/fnins.2023.1223262)
Supplement: Supplementary file 1 [file Data_Sheet_1.pdf]

frontiers

Supplementary Material

Efficient SNN multi-cores MAC array acceleration on SpiNNaker 2

1 DETAILS OF SPLITTING RESULT

Table S1. Splitting result of two echelon algorithms of the balanced random cortex-like network (supplementary for Figure 10).

(a) Echelon (MAC)

processor	core_index	n_rectangles	length	width	length_start	width_start
MAC	1	1	208	520	0	0
	2	2	208	104	0	520
			192	452	8	624
	3	2	192	360	8	1076
			176	224	24	1436
	4	2	176	364	24	1660
			160	272	40	2024
	5	10	160	112	40	2296
			144	264	56	2408
			128	168	72	2672
			112	128	88	2840
			96	76	104	2968
			80	60	120	3044
			64	32	136	3104
			48	20	152	3136
			32	24	168	3156
			16	4	184	3180

(b) Echelon (MAC + ARM)

processor	core_index	n_rectangles	length	width	length_start	width_start
MAC	1	1	192	556	0	0
	2	2	192	68	0	556
			192	484	8	624
	3	2	192	328	8	1108
			176	244	24	1436
	4	2	176	344	24	1680
			160	284	40	2024
	5	10	160	100	40	2308
			144	264	56	2408
			128	168	72	2672
			112	128	88	2840
			96	76	104	2968
			80	60	120	3044
			64	32	136	3104
			48	20	152	3136
			32	24	168	3156
			16	4	184	3180
ARM	1	1	8	125	192	0
	2	1	8	125	192	125
	3	1	8	125	192	250
	4	1	8	125	192	375
	5	1	8	124	192	500

Note: **length** and **width** provide the size of the rectangle to be calculated in the corresponding core with the corresponding processor. **length_start** and **width_start** indicate the absolute starting position of the rectangle in the whole echelon matrix.
